# Simulation of propofol anaesthesia for intracranial decompression using brain hypothermia treatment

**DOI:** 10.1186/1742-4682-4-46

**Published:** 2007-11-29

**Authors:** Lu Gaohua, Hidenori Kimura

**Affiliations:** 1 Bio-Mimetic Control Research Center, The Institute of Physical and Chemical Research (RIKEN) Nagoya, 463-0003, Japan

## Abstract

**Background:**

Although propofol is commonly used for general anaesthesia of normothermic patients in clinical practice, little information is available in the literature regarding the use of propofol anaesthesia for intracranial decompression using brain hypothermia treatment. A novel propofol anaesthesia scheme is proposed that should promote such clinical application and improve understanding of the principles of using propofol anaesthesia for hypothermic intracranial decompression.

**Methods:**

Theoretical analysis was carried out using a previously-developed integrative model of the thermoregulatory, hemodynamic and pharmacokinetic subsystems. Propofol kinetics is described using a framework similar to that of this model and combined with the thermoregulation subsystem through the pharmacodynamic relationship between the blood propofol concentration and the thermoregulatory threshold. A propofol anaesthesia scheme for hypothermic intracranial decompression was simulated using the integrative model.

**Results:**

Compared to the empirical anaesthesia scheme, the proposed anaesthesia scheme can reduce the required propofol dosage by more than 18%.

**Conclusion:**

The integrative model of the thermoregulatory, hemodynamic and pharmacokinetic subsystems is effective in analyzing the use of propofol anaesthesia for hypothermic intracranial decompression. This propofol infusion scheme appears to be more appropriate for clinical application than the empirical one.

## Background

High intracranial pressure (ICP) is still a major cause of mortality in the intensive care unit [1]. Achieving a sustained reduction in ICP in patients with intracranial hypertension remains a great challenge in clinical practice. Brain hypothermia treatment has been demonstrated to be especially effective for patients with refractory intracranial hypertension, for whom conventional therapeutic options for decompression have failed [2]. About half of hypothermia treatments were introduced for the purpose of controlling refractory intracranial hypertension [3].

Besides the management of intracranial temperature and pressure, the administration of anaesthesia is another important task in therapeutic hypothermia treatment. Propofol is widely used in clinical practice for brain hypothermia treatment [4]. However, the rates of propofol administration are based mainly on clinical experience and the normothermic dosage guidelines. An empirical but practical scheme, known as Roberts' step-down infusion, consists of a loading dose of 1 mg kg^-1 ^body weight followed immediately by an infusion of 10 mg kg^-1 ^h^-1 ^for 10 minutes, 8 mg kg^-1 ^h^-1 ^for the next 10 minutes, and 6 mg kg^-1 ^h^-1 ^thereafter [5].

However, the propofol kinetics of hypothermic patients differs significantly from that of normothermic patients because the enzymes that metabolize most drugs are temperature-sensitive [6]. Blood propofol concentrations averaged 28% more at 34°C than at 37°C in healthy volunteers, partially because of the hypothermia-induced decrease in propofol clearance [7]. At the same time, propofol kinetics is clinically affected by hypothermia because therapeutic cooling causes hemodynamic changes. Therefore, a propofol administration scheme used in conjunction with therapeutic cooling should improve the clinical use of propofol anaesthesia for hypothermic intracranial decompression.

The effects of hypothermia on propofol kinetics have not been taken into account theoretically, although some physiologically-based pharmacokinetic (PBPK) models for propofol have been developed recently [8,9]. To the best of our knowledge, theoretical analysis of the use of propofol anaesthesia for hypothermic intracranial decompression is still unavailable in the literature.

On the other hand, an integrative model of the thermoregulatory subsystem, the hemodynamic subsystem and the pharmacokinetic subsystem for a diuretic (mannitol) has been developed for patients undergoing brain hypothermia treatment [10,11]. Hypothermic intracranial decompression was quantitatively characterized by a transfer function [10]. A decoupling control of intracranial temperature and pressure was also established to realize systemic management of cooling and diuresis [11].

We have now used this previously-developed integrative model to analyze the use of propofol anaesthesia for hypothermic intracranial decompression. The pharmacodynamic relationship between the temperature threshold of thermoregulatory reaction (the core temperature triggering vasoconstriction or shivering) and the blood propofol concentration is used to combine the thermoregulatory subsystems with the propofol kinetics. Using the integrative model, a new scheme of propofol anaesthesia for hypothermic intracranial decompression is proposed. Simulations demonstrate the effectiveness of this scheme, and the results suggest that it is more appropriate for clinical application than the empirical Roberts' scheme.

## Model

### Relationship between thermoregulatory threshold and blood propofol concentration

In patients without anaesthesia, vasoconstriction and shivering begin when the core body temperature drops below the thermoregulatory threshold. In brain hypothermia treatment, vasoconstriction is related to high peripheral vascular resistance, inadequate peripheral blood infusion and high mean arterial blood pressure, while shivering increases metabolism and disturbs cardiopulmonary function. Shivering may also cause a transient increase in ICP. Therefore, both vasoconstriction and shivering should be prevented by using anaesthesia during hypothermia treatment.

The duration of action of propofol is short and recovery is rapid because of its rapid distribution and clearance [12]. Compared to other sedatives, propofol provides effective sedation with a more rapid and predictable emergence time for sedation for adults in a variety of clinical settings. Therefore, propofol is widely used in clinical practice. Generally, there is a good correlation between blood propofol concentration and depth of anaesthesia, and continuous infusions of propofol increase the depth in a dose-dependent manner [12].

Matsukawa and colleagues [13] made a systemic investigation of thermoregulation under propofol anaesthesia and found that propofol markedly reduced the vasoconstriction and shivering thresholds. The relationship between the thermoregulatory threshold and propofol concentration in blood can be described mathematically:

*T*_*thres *_= *T*_0*thres *_- *σC*_*artery*_

where *C*_*artery *_(*μ*g ml^-1^) denotes the plasma propofol concentration, which is assumed hereafter to be equal to the blood propofol concentration, *σ *is the slope (*σ *= 0.6°C (*μ*g ml^-1^)^-1 ^for vasoconstriction and 0.7°C (*μ*g ml^-1^)^-1 ^for shivering), *T*_*thres *_(°C) is the thermoregulatory response threshold and *T*_0*thres *_is its initial value. It was estimated that *T*_0*thres *_= 36.5°C for vasoconstriction and 35.6°C for shivering [13].

Because the direct pharmacodynamic effects of propofol on neuroprotection are ignored here for simplification, (1) implies that administration of propofol anaesthesia is unnecessary unless the cooled core temperature, represented here by the brain temperature, is below 36.5°C, the threshold of vasoconstriction. In other words, if the brain temperature is below the temperature threshold determined by the blood propofol concentration, additional propofol should be administered.

The *propofol-threshold mechanism *represented by (1) combines the thermoregulatory subsystem with propofol kinetics pharmacodynamically. It hints at how a patient undergoing hypothermia treatment should be anaesthetized in order to realize stable management of pathophysiological function. If we assume that the brain temperature of a hypothermic patient is slightly (for example, 0.01°C) higher than the temperature threshold determined by the blood propofol concentration, the minimum blood propofol concentration necessary for hypothermic intracranial decompression can be calculated directly using (1).

### Model description

#### Structure and assumptions

**Figure 1:**
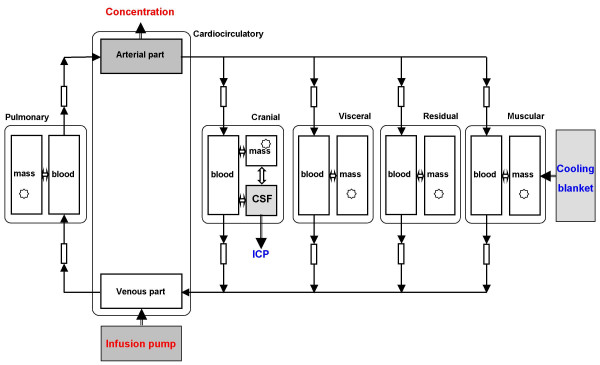
Structure of integrative model. Elevated ICP is reduced by therapeutic cooling [11]. Brain temperature, represented by CSF temperature, is reduced owing to therapeutic cooling. Propofol is administered into venous part of cardiocirculatory segment to achieve the minimum blood propofol concentration needed to inhibit thermoregulatory responses.

The dynamics of the thermoregulatory, hemodynamic and pharmacokinetic subsystems of a patient undergoing brain hypothermia treatment has been modelled previously [10,11]. As shown in Fig. 1, the model is composed of 6 segments or 13 lumped compartments. A cooling blanket is assumed to be applied to the mass compartment of the muscular segment, while the temperature and hydrostatic pressure in the cerebrospinal fluid (CSF) compartment are considered to represent brain temperature and ICP.

Previously, hypothermic effects on the hemodynamics and the pharmacokinetics of diuretic (mannitol) have been considered in order to realize simultaneous control of intracranial temperature and pressure [11]. Here, the thermoregulatory and hemodynamic parts of the integrative model are used without change, while the diuretic kinetic part is changed to describe the propofol kinetics, mainly by changing the pharmacokinetic parameters. Several assumptions are made in modelling the propofol kinetics.

Propofol is administered into the venous compartment and eliminated from the visceral blood compartment. The permeability coefficient of propofol across the vascular wall and the total body clearance are temperature-dependent, as described by the Arrhenius or Van't Hoff equation, as previously reported [10,11].

The main site of propofol action on the thermoregulatory response is the central nervous system. However, the level of anaesthesia has to be estimated from the propofol concentration in the blood, not in the brain mass. Firstly and mainly, this is because the blood propofol concentration is clinically measurable, while measuring the brain propofol concentration in people entails obvious practical and ethical problems. Secondly, the propofol concentration in the CSF can be measured, but it is different in kind from the concentration in brain mass owing to the high protein-binding rate of propofol in brain mass. Moreover, such measurement is still infrequent in clinical practice because accessibility to CSF is limited [14]. Lastly, the *propofol-threshold mechanism *is conveniently based on the blood propofol concentration.

Generally, propofol is associated with good hemodynamic stability although it induces a dose-dependent decrease in systemic vascular resistance, blood pressure and heart rate, together with total body oxygen consumption [12,15]. Hypothermia also reduces the metabolic rate. Therefore, hypothermia and propofol used concurrently have an additive effect on metabolism [16]. For simplicity, however, the direct effects of propofol on brain metabolism and ICP are ignored. Although the hydrostatic pressures of the various compartments vary with respect to therapeutic cooling, it is assumed that vascular resistances are constant during anaesthesia and hypothermia.

#### Governing equation

Because the thermoregulatory and hemodynamic parts of the integrative model are used unchanged, only the propofol kinetics is described (see Appendix). The propofol kinetics is represented systemically by

VdC(t)dt=A(T,P,t)C(t)+u(t),
 MathType@MTEF@5@5@+=feaafiart1ev1aaatCvAUfKttLearuWrP9MDH5MBPbIqV92AaeXatLxBI9gBaebbnrfifHhDYfgasaacPC6xNi=xI8qiVKYPFjYdHaVhbbf9v8qqaqFr0xc9vqFj0dXdbba91qpepeI8k8fiI+fsY=rqGqVepae9pg0db9vqaiVgFr0xfr=xfr=xc9adbaqaaeGacaGaaiaabeqaaeqabiWaaaGcbaGaeCOvayvcfa4aaSaaaeaacqWGKbazcqWHdbWqcqGGOaakcqWG0baDcqGGPaqkaeaacqWGKbazcqWG0baDaaGccqGH9aqpcqWHbbqqcqGGOaakcqWGubavcqGGSaalcqWGqbaucqGGSaalcqWG0baDcqGGPaqkcqWHdbWqcqGGOaakcqWG0baDcqGGPaqkcqGHRaWkcqWH1bqDcqGGOaakcqWG0baDcqGGPaqkcqGGSaalaaa@4A2F@

where **V **∈ *R*^13*x*13 ^is a diagonal matrix corresponding to the distribution volume of propofol in each of the 13 compartments, **C**(*t*) ∈ *R*^13*x*1 ^is the state variable vector of the propofol concentration in each compartment, and **A**(*T*,*P*,*t*) ∈ *R*^13*x*13 ^is a time-varying coefficient matrix determined by both the pharmacokinetic parameters and the physiological states of the thermoregulatory and hemodynamic subsystems. The interactions among the various subsystems are involved in matrix **A **through a temperature-dependent mechanism as well as through blood flow. The input vector, **u**(*t*) ∈ *R*^13*x*1^, represents propofol infusion into the venous compartment.

Combining equation (2) with mathematical descriptions of the thermoregulatory and hemodynamic subsystems produces an integrative model consisting of 39 differential equations. It was programmed in Visual C^++ ^(Version 6.0). Runge-Kutta integration was used to solve these equations numerically.

#### Pharmacokinetic parameters

Data for the integrative model were mainly taken from the literature. The physical and physiological parameters for the thermoregulatory and hemodynamic subsystems are described elsewhere [10,11]. The pharmacokinetic parameters of propofol, including the permeability coefficient, total body clearance, tissue/water partition coefficient, lung sequestration and depth of anaesthesia, are given as follows.

##### Permeability coefficient

The permeability coefficient of propofol across the blood-brain barrier is 0.51 l min^-1 ^[8]. The permeability coefficient across the blood-CSF barrier is assumed to be 5000 times smaller than that across the blood-brain barrier because of the smaller area of the blood-CSF barrier. The permeability coefficients at other extracranial vascular walls are deduced by assuming a vascular permeability comparable to that of the blood-brain barrier. The reference permeability coefficient between the mass and blood compartments is 30.58 l min^-1 ^in the pulmonary segment, 0.14 l min^-1 ^in the visceral segment, 0.53 l min^-1 ^in the muscular segment and 39.27 l min^-1 ^in the residual segment.

The permeability is temperature-dependent and is given by the Arrhenius equation:

k=k0e−E0R(1T+273.15−1T0+273.15)
 MathType@MTEF@5@5@+=feaafiart1ev1aaatCvAUfKttLearuWrP9MDH5MBPbIqV92AaeXatLxBI9gBaebbnrfifHhDYfgasaacPC6xNi=xI8qiVKYPFjYdHaVhbbf9v8qqaqFr0xc9vqFj0dXdbba91qpepeI8k8fiI+fsY=rqGqVepae9pg0db9vqaiVgFr0xfr=xfr=xc9adbaqaaeGacaGaaiaabeqaaeqabiWaaaGcbaGaem4AaSMaeyypa0Jaem4AaS2aaSbaaSqaaiabicdaWaqabaGccqWGLbqzdaahaaWcbeqaaKqbakabgkHiTmaalaaabaGaemyrau0aaSbaaeaacqaIWaamaeqaaaqaaiabdkfasbaacqGGOaakdaWcaaqaaiabigdaXaqaaiabdsfaujabgUcaRiabikdaYiabiEda3iabiodaZiabc6caUiabigdaXiabiwda1aaacqGHsisldaWcaaqaaiabigdaXaqaaiabdsfaunaaBaaabaGaeGimaadabeaacqGHRaWkcqaIYaGmcqaI3aWncqaIZaWmcqGGUaGlcqaIXaqmcqaI1aqnaaGaeiykaKcaaaaa@4C93@

where *k*_0 _(l min^-1^) is the reference propofol permeability at steady-state temperature *T*_0 _(°C), *E*_0 _(kcal mol^-1^) is the Arrhenius activation energy (7 kcal mol^-1 ^in the cranial segment and 5 kcal mol^-1 ^in the other segments [10]), and R is the universal gas constant (1.987 cal mol^-1 ^K^-1^).

##### Total body clearance

Propofol is extensively metabolized and excreted in the urine, mainly as inactive metabolites. Total body clearance ranges from 23–50 ml kg^-1 ^min^-1 ^[12,15]. It is assumed to be discharged from the visceral blood compartment.

Total body clearance is temperature-dependent as the enzymes that metabolize propofol are temperature-sensitive. The Van't Hoff equation is used to describe the temperature dependence of propofol clearance:

e=e0Q10T−T010,
 MathType@MTEF@5@5@+=feaafiart1ev1aaatCvAUfKttLearuWrP9MDH5MBPbIqV92AaeXatLxBI9gBaebbnrfifHhDYfgasaacPC6xNi=xI8qiVKYPFjYdHaVhbbf9v8qqaqFr0xc9vqFj0dXdbba91qpepeI8k8fiI+fsY=rqGqVepae9pg0db9vqaiVgFr0xfr=xfr=xc9adbaqaaeGacaGaaiaabeqaaeqabiWaaaGcbaGaemyzauMaeyypa0Jaemyzau2aaSbaaSqaaiabicdaWaqabaGccqWGrbqudaqhaaWcbaGaeGymaeJaeGimaadajuaGbaWaaSaaaeaacqWGubavcqGHsislcqWGubavdaWgaaqaaiabicdaWaqabaaabaGaeGymaeJaeGimaadaaaaakiabcYcaSaaa@3BEB@

where *e*_0 _(ml kg^-1 ^min^-1^) is the reference propofol clearance at steady-state temperature *T*_0 _(°C), *e*_0 _= 23 ml kg^-1 ^min^-1^, and *Q*_10 _is assumed to be 2 [10].

##### Tissue/water partition coefficient

The data on tissue/water partition coefficients of propofol given by Weaver and colleagues [17] are used: 113.2 for brain mass, 86.2 for visceral mass, 5.3 for pulmonary mass, 51.6 for muscular mass and 35.0 for blood. The CSF/water partition coefficient is assumed to be 1.0. The residual-mass/water partition coefficient is 4700 because of the high fat/water partition coefficient [9].

##### Pulmonary sequestration

Pulmonary sequestration, introduced by Levitt and Schnider [9] in their PBPK model, is considered in this model. The fraction of the dose sequestered by the lungs is 40%, and the time constant of release of this sequestered propofol into the pulmonary blood compartment is 80 min [9].

##### Anaesthesia depth

Clinically relevant blood concentrations of propofol include 1–2 *μ*g ml^-1 ^for long-term sedation in the intensive care unit, at least 2.5 *μ*g ml^-1 ^for satisfactory hypnosis, and 3–11 *μ*g ml^-1 ^for maintenance of satisfactory anaesthesia [18]. The empirical anaesthesia of Roberts' step-down infusion scheme for general anaesthesia in clinical practice targets a blood concentration of 3 *μ*g ml^-1 ^[5]. These data are considered to be a quantitative scale for the depth of propofol anaesthesia.

## Model verification

As the thermoregulatory and hemodynamic parts of the integrative model have been well validated previously [10,11], only the pharmacokinetic part is verified here. Various propofol infusion rates are assumed, and the simulation results for the transient behaviour of the model are compared with published clinical data or theoretical results.

### Cerebrospinal fluid concentration

**Figure 2:**
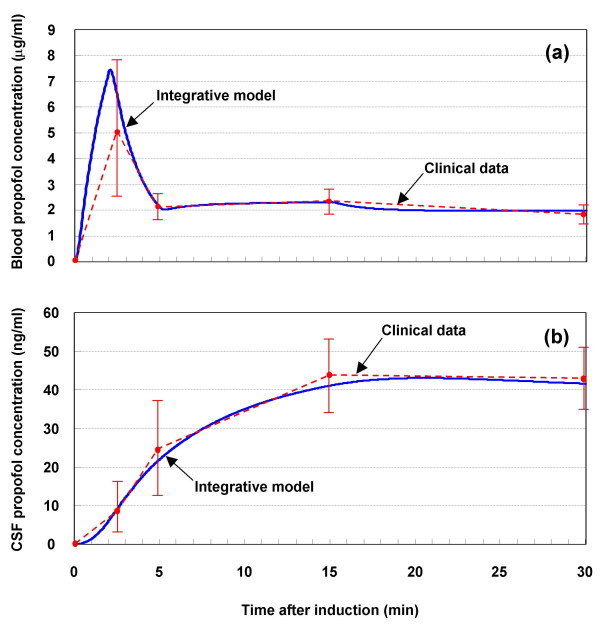
Response of (a) blood and (b) CSF propofol concentration to short-term infusion. Results for pharmacokinetic part of integrative model are compared with clinical data [14].

Engdahl and colleagues [14] measured the propofol concentration in the arterial blood and that in the CSF simultaneously in neurosurgical patients with respect to a step-down propofol infusion. The anaesthesia was induced with a bolus of propofol (2 mg kg^-1^) within 2 min and maintained with a continuous infusion of propofol commencing 5 min after the start of induction at an initial infusion rate of 8 mg kg^-1 ^h^-1 ^for 15 min and then reduced to 6 mg kg^-1 ^h^-1^. A similar manner of propofol infusion is assumed for the pharmacokinetic model. The propofol concentration in the arterial blood and that in the CSF were simulated. The results are shown in Fig. 2; the clinical data of Engdahl and colleagues [14] are also shown for comparison.

The simulated blood propofol concentrations at 2.5, 5, 15 and 30 min were 6.4, 2.1, 2.3 and 2.0 *μ*g ml^-1^, respectively, as shown in Fig. 2(a). The concentration of propofol in the blood increased rapidly during induction. After the bolus was administered, the concentration decreased rapidly. This is consistent with the pharmacokinetics of propofol; that is, its rapid clearance from the blood produces the fast recovery characteristic of the drug. During anaesthesia maintenance, the concentration in the blood increased progressively although it depended on the infusion rate. This reflects the accumulation of propofol in the blood. However, a plateau concentration was reached.

As shown in Fig. 2(b), the simulated CSF propofol concentrations increased during the 30-min simulation. The concentration of propofol in the CSF increased more slowly during induction than it did in the blood. The concentrations at 2.5, 5, 15 and 30 min were 9.1, 22.0, 41.1 and 41.6 ng ml^-1^, respectively. The concentration at 30 min was 2.1% of the blood concentration. These results show that the CSF propofol concentration is positively correlated with, and much lower than, the blood propofol concentration.

All the simulation results agree well with the clinical data. Engdahl and colleagues [14] reported that, for a similar manner of propofol infusion in neurosurgical patients, the blood propofol concentration increased rapidly during induction and reached a plateau concentration (mean 2.24 *μ*g ml^-1^) in about 5 min, which is comparable to our simulation results. In their report, the CSF propofol concentration showed a slower increase during induction and remained almost constant at 35.5 ng ml^-1 ^15–30 min after induction. It was estimated to be 50- to 100-fold lower than that in blood.

Altogether, the blood and CSF propofol concentrations predicted by the model are comparable to the clinical data for short-term (30 min) infusion.

### Arterial blood concentration

Levitt and Schnider [9] developed a PBPK model for propofol and verified it by comparing simulation results with experimental data. The propofol infusion scheme used for both the simulation and clinical experiment was the application of an initial bolus (about 20 s) dose of 2 mg kg^-1 ^body weight followed 60 min later by a 60-min constant infusion at 6 mg kg^-1 ^h^-1^.

The same dosage was assumed for the pharmacokinetic part of the integrative model, and the response of the blood propofol concentration was simulated and compared to the results with the established PBPK model.

**Figure 3:**
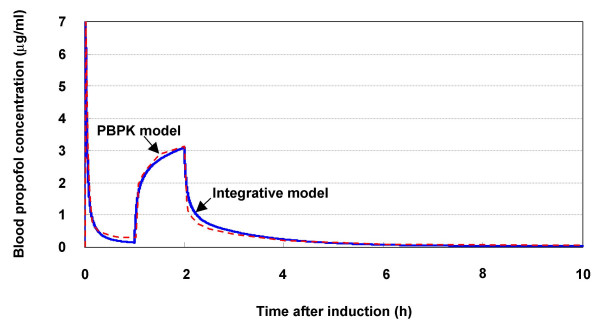
Response of blood propofol concentration to long-term infusion. Results for pharmacokinetic part of integrative model are compared with those for PBPK model [9].

It is observed in Fig. 3 that, in response to a bolus infusion, the blood propofol concentration in the model increased quickly during the injection phase (0–20 s) and reached a peak value of 8.9 *μ*g ml^-1 ^at about 36 s. This is consistent with the clinical observation that propofol action is usually observed within 40 seconds [12]. In contrast, the propofol concentration in brain mass reached a peak value of 8.6 *μ*g ml^-1 ^at about 4 min (data not shown). After the bolus injection, the blood concentration decreased to the eye-opening value (1 *μ*g ml^-1^) at about 8 min and then to 0.15 *μ*g ml^-1 ^at 1 h. Altogether, this impulse-like response of the blood propofol concentration in the integrative model agrees with the theoretical results of the PBPK model.

As shown in Fig. 3, the blood propofol concentration increased progressively during constant propofol infusion for 1–2 h after induction and reached a peak value of about 3.1 *μ*g ml^-1 ^at the end of infusion. It subsequently decreased rapidly. The eye-opening blood concentration (1 *μ*g ml^-1^) was reached about 15 min after the end of infusion. This time is close to that obtained with the PBPK model and that for clinical observation (about 13 min for normal patients) [9].

Altogether, the current model is comparable to the established PBPK model in modelling short-term (0–1 h) and long-term (10 h) propofol kinetics.

## Application of model to propofol anaesthesia

### Scheme for using propofol anaesthesia therapeutically

**Figure 4:**
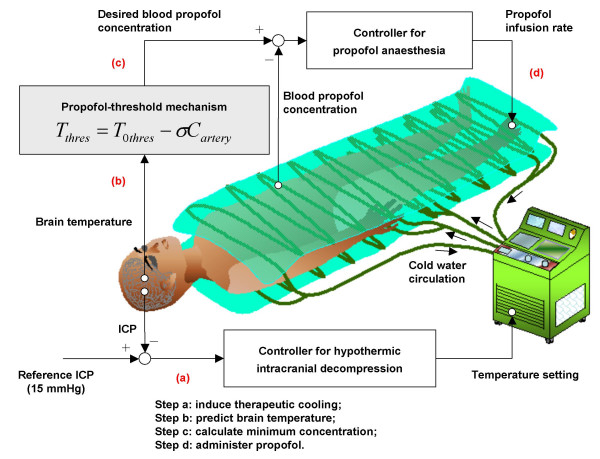
Illustration of proposed scheme for using propofol anaesthesia for hypothermic intracranial decompression: (a) hypothermic intracranial decompression, (b) brain temperature prediction, (c) concentration calculation, (d) propofol administration. The propofol-threshold mechanism is a linear relationship between blood propofol concentration and thermoregulatory threshold.

As shown in Fig. 4, our scheme for using propofol anaesthesia for hypothermic intracranial decompression consists of four steps corresponding to a clinical scenario for automatically regulating propofol administration and therapeutic cooling to control elevated ICP. The proposed scheme is simulated in the integrative model as follows.

#### (a) Hypothermic intracranial decompression

The elevated ICP is decreased by inducing therapeutic cooling. It is simulated in the hemodynamic part of the integrative model using a previously-developed proportional-integral-derivative (PID) feedback temperature controller [11].

#### (b) Brain temperature prediction

The brain temperature is reduced by the therapeutic cooling in Step (a). The cooled brain temperature is predicted using the thermoregulatory part of the integrative model.

#### (c) Concentration calculation

The minimum blood propofol concentration necessary for inhibiting the thermoregulatory response is calculated mathematically using the *propofol-threshold mechanism *of (1). The thermoregulatory threshold determined by the blood propofol concentration is assumed to be slightly (0.01°C in this simulation) below the predicted cooled brain temperature. A *σ *of 0.6°C (*μ*g ml^-1^)^-1 ^and a *T*_0*thres *_of 36.5°C are used in (1) since the blood propofol concentration at which shivering is inhibited is slightly less than that at which vasoconstriction is inhibited.

#### (d) Propofol administration

The simulated rate of propofol administration is controlled by a PID feedback propofol controller so as to realize the minimum blood propofol concentration determined in Step (c). The controller is designed on the basis of the dynamic response of the propofol kinetics corresponding to step-like propofol infusion.

### Preliminary simulation

Prior to simulation of the novel propofol anaesthesia, the integrative model was adjusted to represent a real patient with intracranial hypertension. A PID feedback temperature controller and a PID feedback propofol controller were defined on the basis of the dynamic responses of the integrative model.

#### Model of intracranial hypertension

Various pathophysiological states of elevated ICP have been simulated by adjusting the hemodynamic parameters of the integrative model [10,11]. For example, the absorption rate of CSF from the CSF compartment into the venous compartment could be assumed to be 80% of its normal value. This simulates the presence of a communicating hydrocephalus in clinical practice. Owing to this adjustment, the ICP increased to about 24.5 mmHg [11].

The manipulated model of intracranial hypertension is considered to be the patient in this theoretical discussion. Therapeutic cooling is used to decrease the elevated ICP of the model to 15 mmHg, and propofol anaesthesia for the intracranial decompression is simulated in the proposed and empirical schemes.

#### PID temperature controller

The transient behaviour of the ICP in intracranial hypertension during brain hypothermia treatment was simulated by reducing the cooling temperature from 30 to 29.5°C and then to 29°C [11]. The systemic relationship between the elevated ICP and the cooling temperature is approximated by a linear transfer function:

Ghypot(s)=khypot(1+τhypot1s)(1+τhypot2s)=9.9(1+19.2s)(1+0.3s)(mmHg°C),
 MathType@MTEF@5@5@+=feaafiart1ev1aaatCvAUfKttLearuWrP9MDH5MBPbIqV92AaeXatLxBI9gBaebbnrfifHhDYfgasaacPC6xNi=xI8qiVKYPFjYdHaVhbbf9v8qqaqFr0xc9vqFj0dXdbba91qpepeI8k8fiI+fsY=rqGqVepae9pg0db9vqaiVgFr0xfr=xfr=xc9adbaqaaeGacaGaaiaabeqaaeqabiWaaaGcbaGaem4raC0aaSbaaSqaaiabdIgaOjabdMha5jabdchaWjabd+gaVjabdsha0bqabaGccqGGOaakcqWGZbWCcqGGPaqkcqGH9aqpjuaGdaWcaaqaaiabdUgaRnaaBaaabaGaemiAaGMaemyEaKNaemiCaaNaem4Ba8MaemiDaqhabeaaaeaacqGGOaakcqaIXaqmcqGHRaWkiiGacqWFepaDdaWgaaqaaiabdIgaOjabdMha5jabdchaWjabd+gaVjabdsha0jabigdaXaqabaGaem4CamNaeiykaKIaeiikaGIaeGymaeJaey4kaSIae8hXdq3aaSbaaeaacqWGObaAcqWG5bqEcqWGWbaCcqWGVbWBcqWG0baDcqaIYaGmaeqaaiabdohaZjabcMcaPaaakiabg2da9KqbaoaalaaabaGaeGyoaKJaeiOla4IaeGyoaKdabaGaeiikaGIaeGymaeJaey4kaSIaeGymaeJaeGyoaKJaeiOla4IaeGOmaiJaem4CamNaeiykaKIaeiikaGIaeGymaeJaey4kaSIaeGimaaJaeiOla4IaeG4mamJaem4CamNaeiykaKcaaOGaeiikaGscfa4aaSaaaeaacqWGTbqBcqWGTbqBcqWGibascqWGNbWzaeaacqqGWcaScqWGdbWqaaGccqGGPaqkcqGGSaalaaa@8011@

where *G*_*hypot *_denotes the transfer function from the cooling temperature to ICP, *s *denotes the Laplace operator, *k*_*hypot *_is the static gain (9.9 mmHg°C^-1^), and *τ *_*hypot*1 _and *τ *_*hypot*2 _are time constants (19.2 and 0.3 h, respectively).

**Figure 5:**
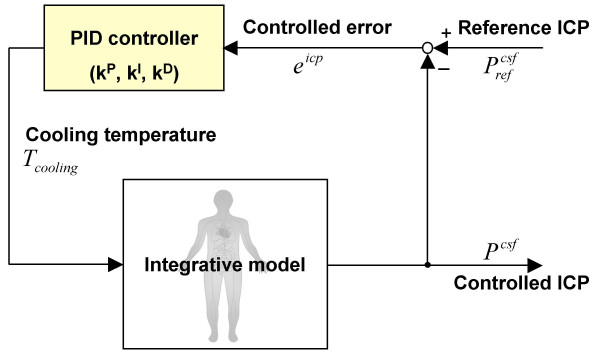
PID feedback temperature controller. *e*^*icp *^is the controlled ICP error (*e*^*icp*^(*t*) = Prefcsf
 MathType@MTEF@5@5@+=feaafiart1ev1aaatCvAUfKttLearuWrP9MDH5MBPbIqV92AaeXatLxBI9gBaebbnrfifHhDYfgasaacPC6xNi=xH8viVGI8Gi=hEeeu0xXdbba9frFj0xb9qqpG0dXdb9aspeI8k8fiI+fsY=rqGqVepae9pg0db9vqaiVgFr0xfr=xfr=xc9adbaqaaeGacaGaaiaabeqaaeqabiWaaaGcbaGaemiuaa1aa0baaSqaaiabdkhaYjabdwgaLjabdAgaMbqaaiabdogaJjabdohaZjabdAgaMbaaaaa@3551@ - *P*^*csf*^(*t*)) and *K*^*P*^, *K*^*I*^, and *K*^*D *^are coefficients for PID feedback control [11].

The PID feedback temperature controller is positioned as shown in Fig. 5.

Tcooling(t)=T0cooling+KhypotP(eicp(t)+1KhypotI∫0teicp(τ)dτ+KhypotDdeicp(t)dt),
 MathType@MTEF@5@5@+=feaafiart1ev1aaatCvAUfKttLearuWrP9MDH5MBPbIqV92AaeXatLxBI9gBaebbnrfifHhDYfgasaacPC6xNi=xI8qiVKYPFjYdHaVhbbf9v8qqaqFr0xc9vqFj0dXdbba91qpepeI8k8fiI+fsY=rqGqVepae9pg0db9vqaiVgFr0xfr=xfr=xc9adbaqaaeGacaGaaiaabeqaaeqabiWaaaGcbaGaemivaq1aaSbaaSqaaiabdogaJjabd+gaVjabd+gaVjabdYgaSjabdMgaPjabd6gaUjabdEgaNbqabaGccqGGOaakcqWG0baDcqGGPaqkcqGH9aqpcqWGubavdaWgaaWcbaGaeGimaaJaem4yamMaem4Ba8Maem4Ba8MaemiBaWMaemyAaKMaemOBa4Maem4zaCgabeaakiabgUcaRiabdUealnaaDaaaleaacqWGObaAcqWG5bqEcqWGWbaCcqWGVbWBcqWG0baDaeaacqWGqbauaaGcdaqadaqaaiabdwgaLnaaCaaaleqabaGaemyAaKMaem4yamMaemiCaahaaOGaeiikaGIaemiDaqNaeiykaKIaey4kaSscfa4aaSaaaeaacqaIXaqmaeaacqWGlbWsdaqhaaqaaiabdIgaOjabdMha5jabdchaWjabd+gaVjabdsha0bqaaiabdMeajbaaaaGcdaWdXaqaaiabdwgaLnaaCaaaleqabaGaemyAaKMaem4yamMaemiCaahaaOGaeiikaGccciGae8hXdqNaeiykaKIaemizaqMae8hXdqhaleaacqaIWaamaeaacqWG0baDa0Gaey4kIipakiabgUcaRiabdUealnaaDaaaleaacqWGObaAcqWG5bqEcqWGWbaCcqWGVbWBcqWG0baDaeaacqWGebaraaqcfa4aaSaaaeaacqWGKbazcqWGLbqzdaahaaqabeaacqWGPbqAcqWGJbWycqWGWbaCaaGaeiikaGIaemiDaqNaeiykaKcabaGaemizaqMaemiDaqhaaaGccaGLOaGaayzkaaGaeiilaWcaaa@9132@

where *T*_*cooling *_(°C) denotes the therapeutic cooling temperature, *T*_0*cooling *_is its normal value (30°C), *e*^*icp *^(mmHg) is the controlled error of ICP (*e*^*icp*^(*t*) = Prefcsf
 MathType@MTEF@5@5@+=feaafiart1ev1aaatCvAUfKttLearuWrP9MDH5MBPbIqV92AaeXatLxBI9gBaebbnrfifHhDYfgasaacPC6xNi=xH8viVGI8Gi=hEeeu0xXdbba9frFj0xb9qqpG0dXdb9aspeI8k8fiI+fsY=rqGqVepae9pg0db9vqaiVgFr0xfr=xfr=xc9adbaqaaeGacaGaaiaabeqaaeqabiWaaaGcbaGaemiuaa1aa0baaSqaaiabdkhaYjabdwgaLjabdAgaMbqaaiabdogaJjabdohaZjabdAgaMbaaaaa@3551@ - *P*^*csf*^(*t*), where Prefcsf
 MathType@MTEF@5@5@+=feaafiart1ev1aaatCvAUfKttLearuWrP9MDH5MBPbIqV92AaeXatLxBI9gBaebbnrfifHhDYfgasaacPC6xNi=xH8viVGI8Gi=hEeeu0xXdbba9frFj0xb9qqpG0dXdb9aspeI8k8fiI+fsY=rqGqVepae9pg0db9vqaiVgFr0xfr=xfr=xc9adbaqaaeGacaGaaiaabeqaaeqabiWaaaGcbaGaemiuaa1aa0baaSqaaiabdkhaYjabdwgaLjabdAgaMbqaaiabdogaJjabdohaZjabdAgaMbaaaaa@3551@ is the reference ICP and Prefcsf
 MathType@MTEF@5@5@+=feaafiart1ev1aaatCvAUfKttLearuWrP9MDH5MBPbIqV92AaeXatLxBI9gBaebbnrfifHhDYfgasaacPC6xNi=xH8viVGI8Gi=hEeeu0xXdbba9frFj0xb9qqpG0dXdb9aspeI8k8fiI+fsY=rqGqVepae9pg0db9vqaiVgFr0xfr=xfr=xc9adbaqaaeGacaGaaiaabeqaaeqabiWaaaGcbaGaemiuaa1aa0baaSqaaiabdkhaYjabdwgaLjabdAgaMbqaaiabdogaJjabdohaZjabdAgaMbaaaaa@3551@ = 15 mmHg), and *K*^*P*^, *K*^*I*^, and *K*^*D *^are PID feedback control coefficients.

KhypotP=τhypot1+τhypot2λhypotkhypot,KhypotI=τhypot1+τhypot2,KhypotD=τhypot1τhypot2τhypot1+τhypot2,
 MathType@MTEF@5@5@+=feaafiart1ev1aaatCvAUfKttLearuWrP9MDH5MBPbIqV92AaeXatLxBI9gBaebbnrfifHhDYfgasaacPC6xNi=xI8qiVKYPFjYdHaVhbbf9v8qqaqFr0xc9vqFj0dXdbba91qpepeI8k8fiI+fsY=rqGqVepae9pg0db9vqaiVgFr0xfr=xfr=xc9adbaqaaeGacaGaaiaabeqaaeqabiWaaaGcbaqbaeqabeWaaaqaaiabdUealnaaDaaaleaacqWGObaAcqWG5bqEcqWGWbaCcqWGVbWBcqWG0baDaeaacqWGqbauaaGccqGH9aqpjuaGdaWcaaqaaGGaciab=r8a0naaBaaabaGaemiAaGMaemyEaKNaemiCaaNaem4Ba8MaemiDaqNaeGymaedabeaacqGHRaWkcqWFepaDdaWgaaqaaiabdIgaOjabdMha5jabdchaWjabd+gaVjabdsha0jabikdaYaqabaaabaGae83UdW2aaSbaaeaacqWGObaAcqWG5bqEcqWGWbaCcqWGVbWBcqWG0baDaeqaaiabdUgaRnaaBaaabaGaemiAaGMaemyEaKNaemiCaaNaem4Ba8MaemiDaqhabeaaaaGccqGGSaalaeaacqWGlbWsdaqhaaWcbaGaemiAaGMaemyEaKNaemiCaaNaem4Ba8MaemiDaqhabaGaemysaKeaaOGaeyypa0Jae8hXdq3aaSbaaSqaaiabdIgaOjabdMha5jabdchaWjabd+gaVjabdsha0jabigdaXaqabaGccqGHRaWkcqWFepaDdaWgaaWcbaGaemiAaGMaemyEaKNaemiCaaNaem4Ba8MaemiDaqNaeGOmaidabeaakiabcYcaSaqaaiabdUealnaaDaaaleaacqWGObaAcqWG5bqEcqWGWbaCcqWGVbWBcqWG0baDaeaacqWGebaraaGccqGH9aqpjuaGdaWcaaqaaiab=r8a0naaBaaabaGaemiAaGMaemyEaKNaemiCaaNaem4Ba8MaemiDaqNaeGymaedabeaacqWFepaDdaWgaaqaaiabdIgaOjabdMha5jabdchaWjabd+gaVjabdsha0jabikdaYaqabaaabaGae8hXdq3aaSbaaeaacqWGObaAcqWG5bqEcqWGWbaCcqWGVbWBcqWG0baDcqaIXaqmaeqaaiabgUcaRiab=r8a0naaBaaabaGaemiAaGMaemyEaKNaemiCaaNaem4Ba8MaemiDaqNaeGOmaidabeaaaaGccqGGSaalaaaaaa@B356@

where *λ *_*hypot *_is an adjustable parameter used to improve the feedback control. In this simulation, *λ *_*hypot *_= 3.5 h.

#### PID propofol controller

The position of the PID feedback propofol controller used to realize the minimum blood propofol concentration is shown in Fig. 4. The PID feedback propofol controller is achieved by simulating the blood propofol concentration response to a constant propofol infusion rate of 1 mg kg^-1 ^h^-1 ^using the pharmacokinetic part of the integrative model. With the help of the System Identification Toolbox of Matlab (version 7.0.4), we use the following transfer function to approximate the dynamic response of blood propofol concentration to propofol infusion:

Gpropl(s)=kpropol(1+τpropl1s)(1+τpropl2s)=0.56(1+1.74s)(1+0.12s)(μg/mlmg/kg/h),
 MathType@MTEF@5@5@+=feaafiart1ev1aaatCvAUfKttLearuWrP9MDH5MBPbIqV92AaeXatLxBI9gBaebbnrfifHhDYfgasaacPC6xNi=xI8qiVKYPFjYdHaVhbbf9v8qqaqFr0xc9vqFj0dXdbba91qpepeI8k8fiI+fsY=rqGqVepae9pg0db9vqaiVgFr0xfr=xfr=xc9adbaqaaeGacaGaaiaabeqaaeqabiWaaaGcbaGaem4raC0aaSbaaSqaaiabdchaWjabdkhaYjabd+gaVjabdchaWjabdYgaSbqabaGccqGGOaakcqWGZbWCcqGGPaqkcqGH9aqpjuaGdaWcaaqaaiabdUgaRnaaBaaabaGaemiCaaNaemOCaiNaem4Ba8MaemiCaaNaem4Ba8MaemiBaWgabeaaaeaacqGGOaakcqaIXaqmcqGHRaWkiiGacqWFepaDdaWgaaqaaiabdchaWjabdkhaYjabd+gaVjabdchaWjabdYgaSjabigdaXaqabaGaem4CamNaeiykaKIaeiikaGIaeGymaeJaey4kaSIae8hXdq3aaSbaaeaacqWGWbaCcqWGYbGCcqWGVbWBcqWGWbaCcqWGSbaBcqaIYaGmaeqaaiabdohaZjabcMcaPaaakiabg2da9KqbaoaalaaabaGaeGimaaJaeiOla4IaeGynauJaeGOnaydabaGaeiikaGIaeGymaeJaey4kaSIaeGymaeJaeiOla4IaeG4naCJaeGinaqJaem4CamNaeiykaKIaeiikaGIaeGymaeJaey4kaSIaeGimaaJaeiOla4IaeGymaeJaeGOmaiJaem4CamNaeiykaKcaaOGaeiikaGscfa4aaSaaaeaacqWF8oqBcqWGNbWzcqGGVaWlcqWGTbqBcqWGSbaBaeaacqWGTbqBcqWGNbWzcqGGVaWlcqWGRbWAcqWGNbWzcqGGVaWlcqWGObaAaaGccqGGPaqkcqGGSaalaaa@8A29@

where *G*_*propl *_denotes the transfer function from the propofol infusion rate to the blood propofol concentration, *k*_*propl *_is the static gain (0.56 *μ*g ml^-1 ^(mg kg^-1 ^h^-1^)^-1^), and *τ *_*propl*1 _and *τ *_*propl*2 _are time constants (1.74 and 0.12 h, respectively).

The static gain, *k*_*propl*_, of the transfer function *G*_*propl*_(s) implies that continuous infusion of propofol at 5–6 mg kg^-1 ^h^-1 ^will result in a blood concentration of about 3 *μ*g ml^-1^. This is consistent with the clinical observation that a blood concentration of 3 *μ*g ml^-1 ^is achieved by tuning the infusion rate to around 6 mg kg^-1 ^h^-1 ^in the empirical Roberts' anaesthesia scheme [5]. Therefore, the estimated transfer function, *G*_*propl*_(s), is considered a reasonable approximation of the propofol kinetics.

On the basis of *G*_*propl*_(s), we developed a PID feedback controller to tune the propofol infusion rate to achieve the target blood propofol concentration.

Ipropl(t)=KproplP(econcn(t)+1KproplI∫0teconcn(τ)dτ+KproplDdeconcn(t)dt)
 MathType@MTEF@5@5@+=feaafiart1ev1aaatCvAUfKttLearuWrP9MDH5MBPbIqV92AaeXatLxBI9gBaebbnrfifHhDYfgasaacPC6xNi=xI8qiVKYPFjYdHaVhbbf9v8qqaqFr0xc9vqFj0dXdbba91qpepeI8k8fiI+fsY=rqGqVepae9pg0db9vqaiVgFr0xfr=xfr=xc9adbaqaaeGacaGaaiaabeqaaeqabiWaaaGcbaGaemysaK0aaSbaaSqaaiabdchaWjabdkhaYjabd+gaVjabdchaWjabdYgaSbqabaGccqGGOaakcqWG0baDcqGGPaqkcqGH9aqpcqWGlbWsdaqhaaWcbaGaemiCaaNaemOCaiNaem4Ba8MaemiCaaNaemiBaWgabaGaemiuaafaaOWaaeWaaeaacqWGLbqzdaahaaWcbeqaaiabdogaJjabd+gaVjabd6gaUjabdogaJjabd6gaUbaakiabcIcaOiabdsha0jabcMcaPiabgUcaRKqbaoaalaaabaGaeGymaedabaGaem4saS0aa0baaeaacqWGWbaCcqWGYbGCcqWGVbWBcqWGWbaCcqWGSbaBaeaacqWGjbqsaaaaaOWaa8qmaeaacqWGLbqzdaahaaWcbeqaaiabdogaJjabd+gaVjabd6gaUjabdogaJjabd6gaUbaakiabcIcaOGGaciab=r8a0jabcMcaPiabdsgaKjab=r8a0bWcbaGaeGimaadabaGaemiDaqhaniabgUIiYdGccqGHRaWkcqWGlbWsdaqhaaWcbaGaemiCaaNaemOCaiNaem4Ba8MaemiCaaNaemiBaWgabaGaemiraqeaaKqbaoaalaaabaGaemizaqMaemyzau2aaWbaaeqabaGaem4yamMaem4Ba8MaemOBa4Maem4yamMaemOBa4gaaiabcIcaOiabdsha0jabcMcaPaqaaiabdsgaKjabdsha0baaaOGaayjkaiaawMcaaaaa@88EC@

KproplP=τpropl1+τpropl2λproplkpropl,KproplI=τpropl1+τpropl2,KproplD=τpropl1τpropl2τpropl1+τpropl2,
 MathType@MTEF@5@5@+=feaafiart1ev1aaatCvAUfKttLearuWrP9MDH5MBPbIqV92AaeXatLxBI9gBaebbnrfifHhDYfgasaacPC6xNi=xI8qiVKYPFjYdHaVhbbf9v8qqaqFr0xc9vqFj0dXdbba91qpepeI8k8fiI+fsY=rqGqVepae9pg0db9vqaiVgFr0xfr=xfr=xc9adbaqaaeGacaGaaiaabeqaaeqabiWaaaGcbaqbaeqabeWaaaqaaiabdUealnaaDaaaleaacqWGWbaCcqWGYbGCcqWGVbWBcqWGWbaCcqWGSbaBaeaacqWGqbauaaGccqGH9aqpjuaGdaWcaaqaaGGaciab=r8a0naaBaaabaGaemiCaaNaemOCaiNaem4Ba8MaemiCaaNaemiBaWMaeGymaedabeaacqGHRaWkcqWFepaDdaWgaaqaaiabdchaWjabdkhaYjabd+gaVjabdchaWjabdYgaSjabikdaYaqabaaabaGae83UdW2aaSbaaeaacqWGWbaCcqWGYbGCcqWGVbWBcqWGWbaCcqWGSbaBaeqaaiabdUgaRnaaBaaabaGaemiCaaNaemOCaiNaem4Ba8MaemiCaaNaemiBaWgabeaaaaGccqGGSaalaeaacqWGlbWsdaqhaaWcbaGaemiCaaNaemOCaiNaem4Ba8MaemiCaaNaemiBaWgabaGaemysaKeaaOGaeyypa0Jae8hXdq3aaSbaaSqaaiabdchaWjabdkhaYjabd+gaVjabdchaWjabdYgaSjabigdaXaqabaGccqGHRaWkcqWFepaDdaWgaaWcbaGaemiCaaNaemOCaiNaem4Ba8MaemiCaaNaemiBaWMaeGOmaidabeaakiabcYcaSaqaaiabdUealnaaDaaaleaacqWGWbaCcqWGYbGCcqWGVbWBcqWGWbaCcqWGSbaBaeaacqWGebaraaGccqGH9aqpjuaGdaWcaaqaaiab=r8a0naaBaaabaGaemiCaaNaemOCaiNaem4Ba8MaemiCaaNaemiBaWMaeGymaedabeaacqWFepaDdaWgaaqaaiabdchaWjabdkhaYjabd+gaVjabdchaWjabdYgaSjabikdaYaqabaaabaGae8hXdq3aaSbaaeaacqWGWbaCcqWGYbGCcqWGVbWBcqWGWbaCcqWGSbaBcqaIXaqmaeqaaiabgUcaRiab=r8a0naaBaaabaGaemiCaaNaemOCaiNaem4Ba8MaemiCaaNaemiBaWMaeGOmaidabeaaaaGccqGGSaalaaaaaa@B2A0@

where *I*_*propl *_(mg kg^-1 ^h^-1^) is the propofol infusion rate, *e*^*concn *^(*μ*g ml^-1^) is the controlled error of the blood propofol concentration (*e*^*concn*^(*t*) = Cdesiredartery
 MathType@MTEF@5@5@+=feaafiart1ev1aaatCvAUfKttLearuWrP9MDH5MBPbIqV92AaeXatLxBI9gBaebbnrfifHhDYfgasaacPC6xNi=xH8viVGI8Gi=hEeeu0xXdbba9frFj0xb9qqpG0dXdb9aspeI8k8fiI+fsY=rqGqVepae9pg0db9vqaiVgFr0xfr=xfr=xc9adbaqaaeGacaGaaiaabeqaaeqabiWaaaGcbaGaem4qam0aa0baaSqaaiabdsgaKjabdwgaLjabdohaZjabdMgaPjabdkhaYjabdwgaLjabdsgaKbqaaiabdggaHjabdkhaYjabdsha0jabdwgaLjabdkhaYjabdMha5baaaaa@3EF2@ - *C*^*artery*^(*t*) , where Cdesiredartery
 MathType@MTEF@5@5@+=feaafiart1ev1aaatCvAUfKttLearuWrP9MDH5MBPbIqV92AaeXatLxBI9gBaebbnrfifHhDYfgasaacPC6xNi=xH8viVGI8Gi=hEeeu0xXdbba9frFj0xb9qqpG0dXdb9aspeI8k8fiI+fsY=rqGqVepae9pg0db9vqaiVgFr0xfr=xfr=xc9adbaqaaeGacaGaaiaabeqaaeqabiWaaaGcbaGaem4qam0aa0baaSqaaiabdsgaKjabdwgaLjabdohaZjabdMgaPjabdkhaYjabdwgaLjabdsgaKbqaaiabdggaHjabdkhaYjabdsha0jabdwgaLjabdkhaYjabdMha5baaaaa@3EF2@ is the target blood propofol concentration, which is calculated from the *propofol-threshold mechanism *represented by (1)), and *λ *_*propl *_= 3.0 min.

### Actual simulation

The proposed scheme for administering propofol anaesthesia for hypothermic intracranial decompression was simulated using the integrative model. For comparison, the empirical scheme of Roberts' step-down propofol infusion, that is, 1 mg kg^-1 ^(0–2 min), 10 mg kg^-1 ^h^-1 ^(2–10 min), 8 mg kg^-1 ^h^-1 ^(10–20 min) and 6 mg kg^-1 ^h^-1 ^(20 min to end of simulation) were also simulated.

## Results

**Figure 6:**
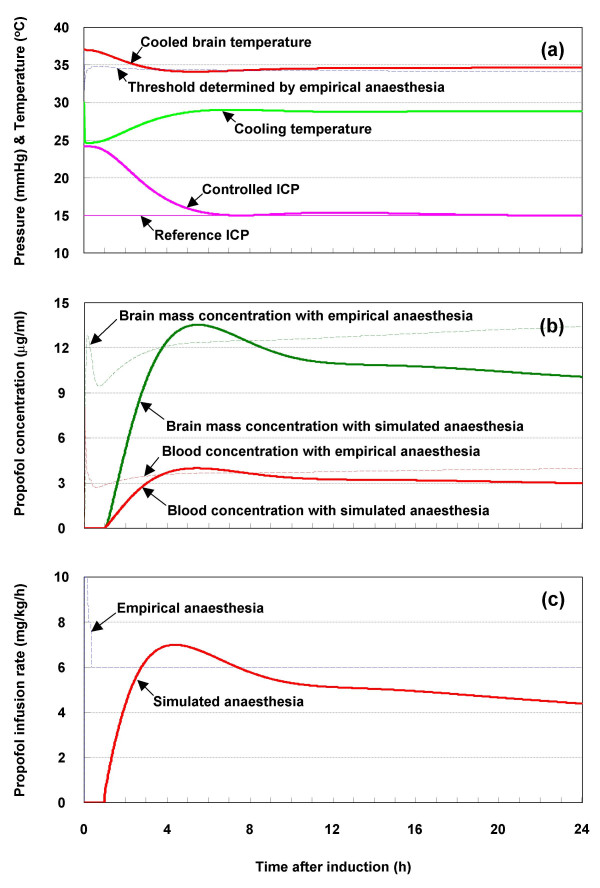
Simultaneous management of intracranial pressure, temperature and anaesthesia: (a) intracranial pressure, brain temperature and cooling temperature, (b) propofol concentration response in blood and brain mass, (c) propofol infusion rate.

As shown in Fig. 6(a), the elevated ICP (24.5 mmHg) was reduced to below 20 mmHg about 2.5 h after inducing the therapeutic cooling and reached the reference ICP (15 mmHg) at about 8 h. The maximum speed of decrease was 2.55 mmHg h^-1 ^at about 2 h. No overshoot of the controlled ICP was observed. These quantitative characteristics depend on the therapeutic cooling temperature determined by the PID feedback temperature controller.

The cooling temperature, which is also shown in Fig. 6(a), was ~25°C at 0.25 h and then increased as the ICP decreased. The highest cooling temperature was ~29°C at about 7 h. The simulated cooling temperature never exceeded the reference value (30°C). This ambient cooling reduced the brain temperature. The static gain of the reduced brain temperature with reference to the cooling temperature was ~2°C°C^-1^, as their values at the end of simulation were 28.9 and 34.7°C, respectively. A cooled brain temperature of 34–35°C corresponds to mild hypothermia, which causes fewer complications than moderate hypothermia (32–33°C) [4]. These results demonstrate that dynamic regulation of the cooling temperature for intracranial decompression is clinically practicable.

The controlled propofol concentrations in the blood and brain mass are shown in Fig. 6(b). During most of the simulated period, the proposed anaesthesia scheme induced propofol concentrations of 3–3.5 *μ*g ml^-1 ^in blood and 10–12 *μ*g ml^-1 ^in brain mass. In contrast, the empirical scheme resulted in propofol concentrations of 3.5–4 *μ*g ml^-1 ^in blood and 12–14 *μ*g ml^-1 ^in brain mass. Therefore, the empirical scheme induces deeper anaesthesia than the proposed scheme. The finding that empirical anaesthesia induces a blood concentration of 3.5–4 *μ*g ml^-1 ^is consistent with clinical observations [5].

As shown in Fig. 6(b), the propofol concentrations in the blood and brain mass were higher over the period 3.7–7.7 h with the proposed scheme than with the empirical scheme. As the controlled brain temperature was much lower during this period, this observation is reasonable. Clinically, a blood propofol concentration of more than 2.5 *μ*g ml^-1 ^is necessary for satisfactory hypnosis and 3–11 *μ*g ml^-1 ^is needed to maintain satisfactory anaesthesia. Therefore, the anaesthesia induced with the proposed scheme, as well as with the empirical scheme, is considered satisfactory.

As shown in Fig. 6(c), the simulated propofol administration varied dynamically in accordance with the cooling temperature. During the first hour, no propofol was necessary although the cooling temperature was somewhat low. This is consistent with the observation that the brain temperature is still higher than the thermoregulatory thresholds during this initial period. In contrast, the infusion rate was high in the first half hour with the empirical scheme.

The total dosage with the proposed scheme was more than 18% less than with the empirical scheme (total dosage of 146.7 mg kg^-1 ^with the empirical scheme and 119.8 mg kg^-1 ^with the proposed one). As pointed out by McKeage and Perry [12], a higher than necessary dosage leads to a higher blood propofol concentration, which may result in a longer recovery time. Therefore, the propofol administration represented by the PID feedback propofol controller is more appropriate for clinical application than the empirical step-down infusion scheme.

The propofol concentrations in the blood and brain mass were higher when the empirical scheme was used with therapeutic cooling than without cooling (data not shown). The temperature threshold with the empirical anaesthesia scheme, as determined in accordance with the *propofol-threshold mechanism *of (1), is shown in Fig. 6(a). It indicates that additional propofol should have been titrated during the 3.7–7.7 h period with the empirical scheme because the cooled brain temperature was below the threshold. Therefore, the total dosage with the empirical scheme would be even larger than with the proposed scheme.

Given the controlled propofol concentration in the blood and brain mass, the lesser depth of anaesthesia and the lower amount of the total dosage, we conclude that the proposed propofol infusion scheme is more appropriate than the empirical scheme.

## Discussion

Brain hypothermia treatment is used for brain-injured patients to protect the brain against secondary neuronal death [4]. It has been shown to reduce elevated ICP effectively in the intensive care unit [2,3]. The major mechanism of intracranial decompression is related to the reduction of cerebral metabolism by therapeutic hypothermia [10,11]. Together with simultaneous management of brain temperature and ICP, adequate anaesthesia is an important component of intensive care. Propofol is widely used in clinical practice, partly because it greatly facilitates management of cardiopulmonary function [4] and partly because it results in a shorter, more predictable emergence time than other sedatives [12]. However, the primary effects of propofol anaesthesia are seldom estimated when it is used for brain hypothermia treatment. In particular, little information is available in the literature on the use of propofol anaesthesia for hypothermic intracranial decompression.

Although two physiologically-based pharmacokinetic (PBPK) models for propofol have recently been developed [8,9], the effects of hypothermia on propofol kinetics have not been considered in the literature. In contrast, we previously developed an integrative model consisting of the thermoregulatory, the hemodynamic and the pharmacokinetic subsystems of a patient under brain hypothermia treatment [10,11]. The interactions among these subsystems are considered via the circulating blood and the temperature-dependence mechanism of various physiological functions such as the metabolism, vascular permeability and drug clearance [11]. By changing its pharmacokinetic part to describe propofol kinetics, we can use this integrative model to simulate the effects of propofol anaesthesia during hypothermia.

The current model is comparable to the PBPK model developed by Levitt and Schnider [9] in modelling short-term (0–1 h) and long-term (10 h) propofol kinetics. However, it is better than the established PBPK model in describing interactions with other physiological functions, such as the thermoregulatory subsystem and the ICP-centered hemodynamics. Furthermore, the pharmacokinetic part of the integrative model describes the tissue and its capillary blood in two adjacent compartments, so it better describes capillary permeability and better represents flow-limited propofol pharmacokinetics.

Compared with the previously-developed integrative model, the present model acquires its originality particularly by introducing the *propofol-threshold mechanism*, which is a pre-established relationship between the blood propofol concentration and the thermoregulatory threshold. Owing to this mechanism, the thermoregulatory subsystem is pharmacodynamically related to the propofol kinetics. That is, a reference blood propofol concentration can be determined to maintain the propofol-determined thermoregulatory threshold below the actual cooled brain temperature.

Using the integrative model and the *propofol-threshold mechanism*, we developed a propofol anaesthesia scheme for hypothermic intracranial decompression. This uses two feedback controllers: a PID feedback temperature controller for tuning the therapeutic cooling and a PID feedback propofol controller for tuning the propofol infusion rate.

As shown by the simulation results, both therapeutic cooling to reduce the elevated ICP and propofol anaesthesia to inhibit the thermoregulatory responses were achieved with the integrative model and the proposed anaesthesia scheme. This proposed scheme is a closed-loop system that facilitates decision-making about propofol administration so as to induce cooling and to reach and maintain a preset target ICP automatically.

As it is still in an initial development stage, the present model depends on some simplification. Therefore, it has some limitations. First, propofol anaesthesia may reduce cerebral metabolism, elevated ICP, cerebral blood flow and cardiac output. However, the pharmacodynamic effects of propofol are ignored. Second, the model depends on several assumptions, including constant vascular resistance, hepatic blood flow, protein and colloid osmotic pressure in the blood, and pulmonary sequestration. Third, the parameters may have a highly dynamic course and differ significantly in importance from one patient to another. Such inter-patient variability is not considered in the present model. By incorporating these physiological mechanisms, we should be able to improve the prediction performance of the model.

Despite these model limitations, our theoretical analysis based on the integrative model has provided useful insights into the simultaneous management of various physiological functions as well as into the automation of medical treatment using closed-loop control techniques. The current theoretical discussion should help improve understanding of the clinical use of propofol anaesthesia for hypothermic intracranial decompression. Given the insights provided by the simulation results, an *in situ *study of the proposed anaesthesia scheme should be fruitful.

## Conclusion

We have used a previously-developed integrative model of the thermoregulatory, hemodynamic and pharmacokinetic subsystems to simulate the use of propofol anaesthesia for hypothermic intracranial decompression. A pharmacodynamic relationship between the blood propofol concentration and the thermoregulatory threshold was introduced to combine the thermoregulation subsystem with the propofol kinetics. A novel scheme for administering propofol anaesthesia was proposed and simulated using the integrative model. Theoretical results suggest that the proposed anaesthesia scheme is more appropriate than the empirical one.

## Appendix

Propofol kinetics is described mathematically on the basis of the mass conservation law. As an example, consider these three equations for the cranial segment.

VcsfdCcsfdt=kBCB(Ccranibloodλblood−Ccsfλcsf)+kbrain(Ccranimassλbrain−Ccsfλcsf)
 MathType@MTEF@5@5@+=feaafiart1ev1aaatCvAUfKttLearuWrP9MDH5MBPbIqV92AaeXatLxBI9gBaebbnrfifHhDYfgasaacPC6xNi=xI8qiVKYPFjYdHaVhbbf9v8qqaqFr0xc9vqFj0dXdbba91qpepeI8k8fiI+fsY=rqGqVepae9pg0db9vqaiVgFr0xfr=xfr=xc9adbaqaaeGacaGaaiaabeqaaeqabiWaaaGcbaGaemOvay1aaWbaaSqabeaacqWGJbWycqWGZbWCcqWGMbGzaaqcfa4aaSaaaeaacqWGKbazcqWGdbWqdaahaaqabeaacqWGJbWycqWGZbWCcqWGMbGzaaaabaGaemizaqMaemiDaqhaaOGaeyypa0Jaem4AaS2aaSbaaSqaaiabdkeacjabdoeadjabdkeacbqabaGccqGGOaakjuaGdaWcaaqaaiabdoeadnaaDaaabaGaem4yamMaemOCaiNaemyyaeMaemOBa4MaemyAaKgabaGaemOyaiMaemiBaWMaem4Ba8Maem4Ba8MaemizaqgaaaqaaGGaciab=T7aSnaaBaaabaGaemOyaiMaemiBaWMaem4Ba8Maem4Ba8MaemizaqgabeaaaaGccqGHsisljuaGdaWcaaqaaiabdoeadnaaCaaabeqaaiabdogaJjabdohaZjabdAgaMbaaaeaacqWF7oaBdaWgaaqaaiabdogaJjabdohaZjabdAgaMbqabaaaaOGaeiykaKIaey4kaSIaem4AaS2aaSbaaSqaaiabdkgaIjabdkhaYjabdggaHjabdMgaPjabd6gaUbqabaGccqGGOaakjuaGdaWcaaqaaiabdoeadnaaDaaabaGaem4yamMaemOCaiNaemyyaeMaemOBa4MaemyAaKgabaGaemyBa0MaemyyaeMaem4CamNaem4Camhaaaqaaiab=T7aSnaaBaaabaGaemOyaiMaemOCaiNaemyyaeMaemyAaKMaemOBa4gabeaaaaGccqGHsisljuaGdaWcaaqaaiabdoeadnaaCaaabeqaaiabdogaJjabdohaZjabdAgaMbaaaeaacqWF7oaBdaWgaaqaaiabdogaJjabdohaZjabdAgaMbqabaaaaOGaeiykaKcaaa@9648@

VcranimassdCcranimassdt=kBBB(Ccranibloodλblood−Ccranimassλbrain)+kbrain(Ccsfλcsf−Ccranimassλbrain)
 MathType@MTEF@5@5@+=feaafiart1ev1aaatCvAUfKttLearuWrP9MDH5MBPbIqV92AaeXatLxBI9gBaebbnrfifHhDYfgasaacPC6xNi=xI8qiVKYPFjYdHaVhbbf9v8qqaqFr0xc9vqFj0dXdbba91qpepeI8k8fiI+fsY=rqGqVepae9pg0db9vqaiVgFr0xfr=xfr=xc9adbaqaaeGacaGaaiaabeqaaeqabiWaaaGcbaGaemOvay1aa0baaSqaaiabdogaJjabdkhaYjabdggaHjabd6gaUjabdMgaPbqaaiabd2gaTjabdggaHjabdohaZjabdohaZbaajuaGdaWcaaqaaiabdsgaKjabdoeadnaaDaaabaGaem4yamMaemOCaiNaemyyaeMaemOBa4MaemyAaKgabaGaemyBa0MaemyyaeMaem4CamNaem4CamhaaaqaaiabdsgaKjabdsha0baakiabg2da9iabdUgaRnaaBaaaleaacqWGcbGqcqWGcbGqcqWGcbGqaeqaaOGaeiikaGscfa4aaSaaaeaacqWGdbWqdaqhaaqaaiabdogaJjabdkhaYjabdggaHjabd6gaUjabdMgaPbqaaiabdkgaIjabdYgaSjabd+gaVjabd+gaVjabdsgaKbaaaeaaiiGacqWF7oaBdaWgaaqaaiabdkgaIjabdYgaSjabd+gaVjabd+gaVjabdsgaKbqabaaaaOGaeyOeI0scfa4aaSaaaeaacqWGdbWqdaqhaaqaaiabdogaJjabdkhaYjabdggaHjabd6gaUjabdMgaPbqaaiabd2gaTjabdggaHjabdohaZjabdohaZbaaaeaacqWF7oaBdaWgaaqaaiabdkgaIjabdkhaYjabdggaHjabdMgaPjabd6gaUbqabaaaaOGaeiykaKIaey4kaSIaem4AaS2aaSbaaSqaaiabdkgaIjabdkhaYjabdggaHjabdMgaPjabd6gaUbqabaGccqGGOaakjuaGdaWcaaqaaiabdoeadnaaCaaabeqaaiabdogaJjabdohaZjabdAgaMbaaaeaacqWF7oaBdaWgaaqaaiabdogaJjabdohaZjabdAgaMbqabaaaaOGaeyOeI0scfa4aaSaaaeaacqWGdbWqdaqhaaqaaiabdogaJjabdkhaYjabdggaHjabd6gaUjabdMgaPbqaaiabd2gaTjabdggaHjabdohaZjabdohaZbaaaeaacqWF7oaBdaWgaaqaaiabdkgaIjabdkhaYjabdggaHjabdMgaPjabd6gaUbqabaaaaOGaeiykaKcaaa@B1B8@

VcraniblooddCcraniblooddt=kBCB(Ccsfλcsf−Ccranibloodλblood)+kBBB(Ccranimassλbrain−Ccranibloodλblood)+wcraniarteryCartery−wcraniveinCcraniblood,
 MathType@MTEF@5@5@+=feaafiart1ev1aaatCvAUfKttLearuWrP9MDH5MBPbIqV92AaeXatLxBI9gBaebbnrfifHhDYfgasaacPC6xNi=xI8qiVKYPFjYdHaVhbbf9v8qqaqFr0xc9vqFj0dXdbba91qpepeI8k8fiI+fsY=rqGqVepae9pg0db9vqaiVgFr0xfr=xfr=xc9adbaqaaeGacaGaaiaabeqaaeqabiWaaaGcbaGaemOvay1aa0baaSqaaiabdogaJjabdkhaYjabdggaHjabd6gaUjabdMgaPbqaaiabdkgaIjabdYgaSjabd+gaVjabd+gaVjabdsgaKbaajuaGdaWcaaqaaiabdsgaKjabdoeadnaaDaaabaGaem4yamMaemOCaiNaemyyaeMaemOBa4MaemyAaKgabaGaemOyaiMaemiBaWMaem4Ba8Maem4Ba8MaemizaqgaaaqaaiabdsgaKjabdsha0baakiabg2da9iabdUgaRnaaBaaaleaacqWGcbGqcqWGdbWqcqWGcbGqaeqaaOGaeiikaGscfa4aaSaaaeaacqWGdbWqdaahaaqabeaacqWGJbWycqWGZbWCcqWGMbGzaaaabaacciGae83UdW2aaSbaaeaacqWGJbWycqWGZbWCcqWGMbGzaeqaaaaakiabgkHiTKqbaoaalaaabaGaem4qam0aa0baaeaacqWGJbWycqWGYbGCcqWGHbqycqWGUbGBcqWGPbqAaeaacqWGIbGycqWGSbaBcqWGVbWBcqWGVbWBcqWGKbazaaaabaGae83UdW2aaSbaaeaacqWGIbGycqWGSbaBcqWGVbWBcqWGVbWBcqWGKbazaeqaaaaakiabcMcaPiabgUcaRiabdUgaRnaaBaaaleaacqWGcbGqcqWGcbGqcqWGcbGqaeqaaOGaeiikaGscfa4aaSaaaeaacqWGdbWqdaqhaaqaaiabdogaJjabdkhaYjabdggaHjabd6gaUjabdMgaPbqaaiabd2gaTjabdggaHjabdohaZjabdohaZbaaaeaacqWF7oaBdaWgaaqaaiabdkgaIjabdkhaYjabdggaHjabdMgaPjabd6gaUbqabaaaaOGaeyOeI0scfa4aaSaaaeaacqWGdbWqdaqhaaqaaiabdogaJjabdkhaYjabdggaHjabd6gaUjabdMgaPbqaaiabdkgaIjabdYgaSjabd+gaVjabd+gaVjabdsgaKbaaaeaacqWF7oaBdaWgaaqaaiabdkgaIjabdYgaSjabd+gaVjabd+gaVjabdsgaKbqabaaaaOGaeiykaKIaey4kaSIaem4DaC3aa0baaSqaaiabdogaJjabdkhaYjabdggaHjabd6gaUjabdMgaPbqaaiabdggaHjabdkhaYjabdsha0jabdwgaLjabdkhaYjabdMha5baakiabdoeadnaaCaaaleqabaGaemyyaeMaemOCaiNaemiDaqNaemyzauMaemOCaiNaemyEaKhaaOGaeyOeI0Iaem4DaC3aa0baaSqaaiabdogaJjabdkhaYjabdggaHjabd6gaUjabdMgaPbqaaiabdAha2jabdwgaLjabdMgaPjabd6gaUbaakiabdoeadnaaDaaaleaacqWGJbWycqWGYbGCcqWGHbqycqWGUbGBcqWGPbqAaeaacqWGIbGycqWGSbaBcqWGVbWBcqWGVbWBcqWGKbazaaGccqGGSaalaaa@EBF0@

where VXY
 MathType@MTEF@5@5@+=feaafiart1ev1aaatCvAUfKttLearuWrP9MDH5MBPbIqV92AaeXatLxBI9gBaebbnrfifHhDYfgasaacPC6xNi=xH8viVGI8Gi=hEeeu0xXdbba9frFj0xb9qqpG0dXdb9aspeI8k8fiI+fsY=rqGqVepae9pg0db9vqaiVgFr0xfr=xfr=xc9adbaqaaeGacaGaaiaabeqaaeqabiWaaaGcbaGaemOvay1aa0baaSqaaiabdIfaybqaaiabdMfazbaaaaa@2FA9@ and CXY
 MathType@MTEF@5@5@+=feaafiart1ev1aaatCvAUfKttLearuWrP9MDH5MBPbIqV92AaeXatLxBI9gBaebbnrfifHhDYfgasaacPC6xNi=xH8viVGI8Gi=hEeeu0xXdbba9frFj0xb9qqpG0dXdb9aspeI8k8fiI+fsY=rqGqVepae9pg0db9vqaiVgFr0xfr=xfr=xc9adbaqaaeGacaGaaiaabeqaaeqabiWaaaGcbaGaem4qam0aa0baaSqaaiabdIfaybqaaiabdMfazbaaaaa@2F83@ denote the distributed volume and propofol concentration in compartment *Y *of segment *X*, *k*_*BCB *_and *k*_*BBB *_are the permeability coefficient at the blood-CSF barrier and blood-brain barrier, respectively, *k*_*brain *_is the permeability coefficient between the brain mass and CSF, wcraniartery
 MathType@MTEF@5@5@+=feaafiart1ev1aaatCvAUfKttLearuWrP9MDH5MBPbIqV92AaeXatLxBI9gBaebbnrfifHhDYfgasaacPC6xNi=xH8viVGI8Gi=hEeeu0xXdbba9frFj0xb9qqpG0dXdb9aspeI8k8fiI+fsY=rqGqVepae9pg0db9vqaiVgFr0xfr=xfr=xc9adbaqaaeGacaGaaiaabeqaaeqabiWaaaGcbaGaem4DaC3aa0baaSqaaiabdogaJjabdkhaYjabdggaHjabd6gaUjabdMgaPbqaaiabdggaHjabdkhaYjabdsha0jabdwgaLjabdkhaYjabdMha5baaaaa@3CA2@ represents the blood flow from the arterial compartment into the cranial blood compartment, wcranivein
 MathType@MTEF@5@5@+=feaafiart1ev1aaatCvAUfKttLearuWrP9MDH5MBPbIqV92AaeXatLxBI9gBaebbnrfifHhDYfgasaacPC6xNi=xH8viVGI8Gi=hEeeu0xXdbba9frFj0xb9qqpG0dXdb9aspeI8k8fiI+fsY=rqGqVepae9pg0db9vqaiVgFr0xfr=xfr=xc9adbaqaaeGacaGaaiaabeqaaeqabiWaaaGcbaGaem4DaC3aa0baaSqaaiabdogaJjabdkhaYjabdggaHjabd6gaUjabdMgaPbqaaiabdAha2jabdwgaLjabdMgaPjabd6gaUbaaaaa@39C6@ represents the blood flow from the cranial blood compartment into the venous compartment, *λ *_*csf*_, *λ *_*blood *_and *λ *_*brain *_are the CSF/water, blood/water and brain/water partition coefficients of propofol, respectively.

Thirteen differential equations of propofol concentrations are available for describing the pharmacokinetics of propofol.

## Competing interests

The author(s) declare that they have no competing interests.
